# Advance and Application of Single-cell Transcriptomics in Auditory Research

**DOI:** 10.1007/s12264-023-01149-z

**Published:** 2023-11-28

**Authors:** Xiangyu Ma, Jiamin Guo, Mengyao Tian, Yaoyang Fu, Pei Jiang, Yuan Zhang, Renjie Chai

**Affiliations:** 1 State Key Laboratory of Digital Medical Engineering, Department of Otolaryngology Head and Neck Surgery, Zhongda Hospital, School of Life Sciences and Technology, Advanced Institute for Life and Health, Jiangsu Province High-Tech Key Laboratory for Bio-Medical Research, Southeast University, Nanjing, 210096 China; 2 Department of Psychiatry, Affiliated Hangzhou First People’s Hospital, Zhejiang University school of Medicine, Hangzhou, 310030 China; 3 Department of Otolaryngology Head and Neck Surgery, Affiliated Drum Tower Hospital of Nanjing University Medical School, Jiangsu Provincial Key Medical Discipline (Laboratory), Nanjing, China; 4 Research Institute of Otolaryngology, Nanjing, 210008 China; 5 Department of Otolaryngology Head and Neck Surgery, Sichuan Provincial People’s Hospital, University of Electronic Science and Technology of China, Chengdu, 610072 China; 6 Co-Innovation Center of Neuroregeneration, Nantong University, Nantong, 226001 China; 7 Institute for Stem Cell and Regeneration, Chinese Academy of Science, Beijing, 101408 China; 8 Beijing Key Laboratory of Neural Regeneration and Repair, Capital Medical University, Beijing, 100069 China

**Keywords:** Single-cell RNA sequencing, Inner ear, Cochlear, Auditory sensory epithelium, Spiral ganglion neuron

## Abstract

Hearing loss and deafness, as a worldwide disability disease, have been troubling human beings. However, the auditory organ of the inner ear is highly heterogeneous and has a very limited number of cells, which are largely uncharacterized in depth. Recently, with the development and utilization of single-cell RNA sequencing (scRNA-seq), researchers have been able to unveil the complex and sophisticated biological mechanisms of various types of cells in the auditory organ at the single-cell level and address the challenges of cellular heterogeneity that are not resolved through by conventional bulk RNA sequencing (bulk RNA-seq). Herein, we reviewed the application of scRNA-seq technology in auditory research, with the aim of providing a reference for the development of auditory organs, the pathogenesis of hearing loss, and regenerative therapy. Prospects about spatial transcriptomic scRNA-seq, single-cell based genome, and Live-seq technology will also be discussed.

## Introduction

Hearing plays a vital role in social communication, but ~ 5% of the global population suffers from disabling hearing loss at present [1]. Therefore, elucidating the mechanism of hearing loss is essential for the treatment and prevention of hearing impairment and deafness [2, 3]. Studies have indicated that the cochlea, the spiral ganglion neuron (SGN), and the endolymphatic sac are crucial for auditory signals and function, especially the auditory sensory epithelium in the cochlea exhibit a high degree of heterogeneity [4], which is a complexly structured tissue composed of two types of hair cells (HCs) (outer hair cells (OHCs) and inner hair cells (IHCs)) and diverse supporting cells (SCs). Additionally, hearing loss involves a complex series of molecular and cellular events that lead to cell death and tissue damage.

**Fig. 1:**
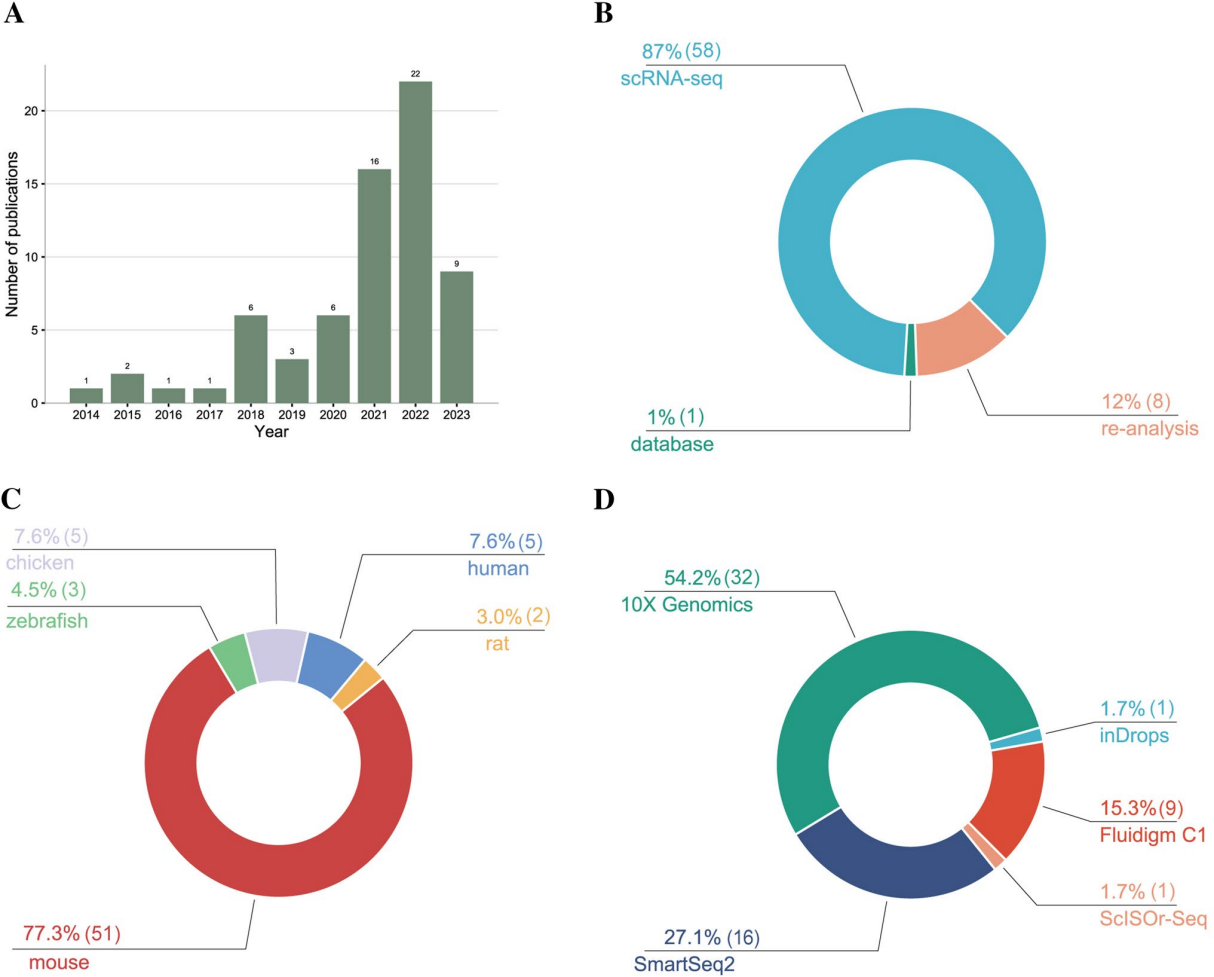
The summary of publications of scRNA-seq in auditory research. **A** A stack histogram shows that between 2014 and July 29, 2023, the number of publications related to single-cell transcriptomics in the field of auditory research has increased, and the number has increased dramatically in the last two years. **B** Number of publications by study types."scRNA-seq" indicates the original studies that generate scRNA-seq data."re-analysis refers to the re-analysis studies of the published scRNA-seg datasets."database" means the online database dedicated to the study of the inner ear. **C** Number of publications by type of source of sample species. **D** Number of publications by sequencing method types.

The conventional bulk RNA-seq can only sequence a group of cells, which may lead to data distortion due to an inability to capture single cell-specific RNA expression, thereby hindering significantly the study of highly heterogeneous organs or tissues, especially the cochlea. In contrast, scRNA-seq offers a powerful method for unbiased, high-throughput, and high-resolution analysis of transcriptome at the single-cell level, allowing for a better understanding of transcriptional features of individual cells in specific contexts, particularly in the fields of development and disease [5–7]. Herein, we briefly overview the existing scRNA-seq technology and its corresponding characteristics and meticulously summarize its diverse applications in the field of cochlear research (Fig. 1A–C), as well as discuss the potential developments and applications of scRNA-seq technology in auditory research.

## Single-cell RNA Sequencing Technology

### Development of scRNA-seq Technology

Since scRNA-seq was initially reported by Tang *et al.* [8], it has greatly facilitated the transformative investigation into biology across multiple fields [9]. With advances in sequencing technology and decreasing sequencing costs, various scRNA-seq methods and commercial platforms have emerged. In the field of inner ear and auditory, Smart-seq (Fluidigm C1 system) [10], Smart-seq 2 [11], and Drop-seq (10X Genomics Chromium) [12] are the most widely used methods (Fig. 1D). The Fluidigm C1 system can capture full-length mRNAs with high transcriptome coverage and detect single nucleotide polymorphisms and mutations by using Smart-seq method, albeit with relatively low throughput. The Smart-seq 2 is an improved and optimized version of Smart-seq but has not yet been commercialized. The 10X Genomics Chromium, another commercial sequencing platform established on Drop-seq, has high cell throughput but lower sequencing depth [13].

### Current Workflow and Tools for scRNA-seq Technology

**Fig. 2:**
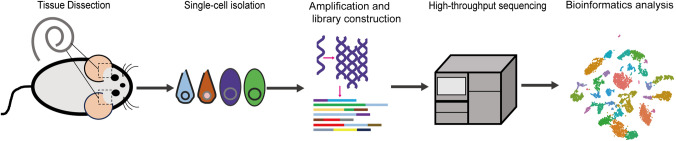
Basic process of scRNA-seq.

The main steps of scRNA-seq include single-cell isolation, amplification and library construction, high-throughput sequencing, and bioinformatics analysis (Fig. 2). The main methods of single-cell isolation are fluorescence-activated cell sorting (FACS) [14], magnetic-activated cell sorting (MACS) [15], patch clamp suction [8], and microfluidic chip capture [16]. FACS and MACS have the advantages of sorting a large number of cells and rapid quantitative analysis. Patch-clamp suction can select specific cells under the microscope based on morphology to further sequence, making it suitable for rare and well-defined cell types, such as auditory HCs and SCs. Microfluidic chip capture has the advantage of using a smaller volume of cell suspension and reducing the risk of external contamination. Meanwhile, tools of related bioinformatics analysis have been developed, such as Seurat, Monocle, Scanpy, Linnorm, and Monocle [17], which allows us to conduct comprehensive analyses of single-cell transcriptome data, such as (1) cell type clustering annotation and differential expression analysis (e.g. Seurat [18]), (2) transcription factor (TF) analysis (e.g. SCENIC [19]), (3) pseudo-time sequence analysis (e.g. Monocle 2 [20]), (4) cell-cell communication analysis (e.g. CellPhoneDB [21]), and so on. Remarkably, some visualization databases, such as the gEAR database [22], can provide convenient access to scRNA-seq data in the field of auditory system research.

## Application of scRNA-seq in Auditory Sensory Epithelium

### Auditory Epithelial Cell Heterogeneity

In 2015, Burns *et al.* used scRNA-seq to analyze P1 cochlear epithelial tissue and divide it into four cell populations: HCs, SCs, medial non-sensory cells, and lateral non-sensory cells [23], especially identified new marker genes for HC (*Rasd2*, *Anxa4*, and *Pcp4*), medial SC (*Cdh4* and *Mia1*), and lateral SC (*Cntn1*). Hoa *et al.* further divided adult mouse SCs into two subgroups (medial SC and lateral SC) and found marker genes for medial SC (*S100a6* and *Pla2g7*) and lateral SC (*Tuba1b* and *Spry2*) [24]. Interestingly, S-phase genes (e.g. *Mcm4*), G2/M phase genes (*Cdk1* and *Mki67*), and genes enriched in Lgr5+ cells in the neonatal cochlea (*Cdkn1b* and *Shc3*) were highly expressed in the lateral SC subgroup, suggesting that lateral SCs may contribute to HC regeneration in the adult inner ear. Waldhaus *et al.* characterized the transcriptomes of various cell types in the neonatal auditory sensory epithelium, finding that inner pillar cells (IPCs) in SCs tend to regenerate HCs accompanied by significantly expressed genes in Notch signaling pathway (*Hes5*, *Hey1,* and *Lfng*), Wnt signaling pathway (*Fzd6* and *Lgr5*), and early cell cycle (*Ccnd1* and *Cdk6*), especially HC differentiation-related genes (s *Otof*, *Ptprq*, and *Clrn1*) and tonotopic axis establishment-related genes (*Actb*, *Pcdh15*) showed a gradient expression trend [25].

Ranum *et al.* compared the transcriptomes of IHCs and OHCs in mouse cochlea using scRNA-seq [26], finding that Calcium-related genes *Ocm* and *Sri* are the top-ranking expression in OHCs, and Sorcin (encoded by *Sri*) is a specific expression in OHCs, implying that OHCs have unique requirements for calcium compared to IHCs. Notably, taking advantage of SmartSeq2, they further detected 20 highly conserved unannotated exons (e.g. *Cabp2* exon 1B, *Cacna1d* exon 1B, and *Coch* exon 3B) in 12 deafness-related genes, and discovered five subtypes of *Cabp2* in 12 OHCs by using nanopore single-cell full-length transcriptome sequencing technology, which may have important implications for revealing the genetic basis of deafness.

What’s more, Qian *et al.* used scRNA-seq to characterize macula hair cells (MHCs) of newborn zebrafish and found that roles of many highly expressed genes have not been reported in HCs, but the loss of *mb* caused impairment and dysfunction of HCs [27]. Janesick *et al.* further identified some highly expressed genes in chicken cochlear tall HCs (e.g. *Ccl14*, *Grand3,* and *Kcnab1*) and many specifically expressed genes in short HCs (e.g. *C14orf180*, *Sdr42e2,* and *Zdhhc23*) [28]. These studies also discovered a marker gene *Itm2c* for superior tall HCs, *Lcat*, *Glipr1l,* and *Dkk3* for medial SCs, and *Ntn4l*, *Smoc2* and *Timp3* for lateral SCs. Meanwhile, they also characterized a series of other types of cells, such as far medial clear cells (*Podxl*), far lateral hyaline cells (*Apoa1*), and so on.

### Auditory Epithelial Cell Development and Aging

Of note, Wang *et al.* integrated scATAC-seq and scRNA-seq data from P2 mice cochleae to identify a regulatory network composed of 20 HC-specific TFs [29]. Among 20 TFs, *Zbtb18* participated in sensorineural hearing loss (SNHL) [30], *Insm1*, *Foxo4, Tcf4,* and *Glis3* were involved in controlling the differentiation of IHCs and OHCs. Interestingly, Kolla *et al.* mapped the single-cell atlas of the early development (E14, E16, P1, P7) of the cochlea and found that OHCs and their surrounding SCs originate from lateral prosensory cells (LPsCs) in the fate map of progenitor cells [31], especially *Tgfβr1* associated with hearing loss [32] was detected only in LPsCs. Furthermore, Xu *et al.* further refined the single-cell atlas of the cochlea during development and maturation (P7, P14, P28) [33], and found that the maturation of IHCs peaked at P14 but OHCs continued to develop until P28, as well as the dynamic expression of *Mia* and *Pcp4,* existed during cochlear HC maturation. Jean *et al.* also conducted scRNA-seq on over 120,000 cells from three key differentiation stages, namely P8 (before hearing onset), P12 (upon hearing onset), and P20 (ear cochlear maturation), to establish a comprehensive transcriptomic atlas of the mouse cochlea [34]. They identified three previously unknown cell types, two of which comprised the modiolus (*Aldh1a2* and *Slc7a11*), a cell type housing the major auditory neurons and blood vessels. The third cell type (*Fxyd2* and *Kcnk2*) was found to be involved in the development of the vestibular stage and was named the scala vestibuli border (SVB) cells. Furthermore, they analyzed the expression patterns of deafness-related genes and crucial genes involved in cochlear development and function.

Type 2 deiodinase (Dio2), as a thyroid hormone-activating enzyme, is crucial for the maturation and auditory development of the cochlea [35]. *Dio2*-deficient mice exhibit deafness and cochlear defects [36], but the low and transient expression of *Dio2* made it difficult to identify the specific cell types expressing *Dio2* in the cochlea. Ng *et al.* identified two major cell types expressing *Dio2* in mouse cochlea by performing scRNA-seq on Dio2+ cells, including fibrocytes (*Sox9*, *Tbx18,* and *Gjb2*) and osteoblasts (*Runx2*, *Sp7*, and *Tmem119*) [37], and found that most fibrocytes express candidate membrane transporters of thyroid hormone *Slco1c1*, *Slc16a2* and *Slco1a4*, while osteoblasts specifically highly express *Slco3a1*.

Chessum *et al.* found that *Ikzf2*, encoding TF Helios, is required for the hearing and functional maturation of mouse OHCs[38]. They performed scRNA-seq of mouse HCs that overexpressed *Ikzf2* and found that the overexpression of *Ikzf2* in IHCs led to the upregulation of OHC-specific genes (*Ocm*, *Pde6d*, *Ldhb,* and *Lbh*) and the downregulation of IHC-specific genes (*Slc17a8*, *Atp2a3* and *Fgf8*). Bi *et al.* compared the scRNA-seq data of OHCs and IHCs in the mouse cochlea to identify essential genes for IHC development [39] and found that *Tbx2* as a TF was abundantly expressed specifically in IHCs, but hardly expressed in OHCs, especially knockout experiments revealed that IHCs tended to differentiate into OHCs when *Tbx2* was absent. Interestingly, integrating and analyzing the previously published single-cell transcriptome data [31, 40] on OHCs at different stages found that induced-OHCs after the loss of *Tbx2* shared the most similarity with OHCs from P30 WT mice.

In invertebrate animal models, retinoic acid (RA) has been demonstrated to be pivotal in the fate of the otic placode, as demonstrated by Saeki *et al.* in a study [41]. They examined the function of the RA signaling pathway in mammalian inner ear development using scRNA-seq of human otic placode-like organ cells treated with and without RA, finding that RA affected the expression of several marker genes (*Six1*, *Eya1*, *Pax2*, and *Gata3*) in some anterior otocysts. Moreover, Moore *et al.* employed pharmacological manipulation using the small molecule purmoramine (PUR), an agonist of SHH and IWP2 (an inhibitor of WNT signaling) to modulate the expression of SHH and WNT signaling in otic progenitor cells [42]. This manipulation resulted in the upregulation of ventral genes in the developing otic epithelium, such as *Otx1/2*, *Nr2f1/2*, *Edn3*, and *Rspo3*. Subsequently, derived HCs in human otic organoids exhibited molecular characteristics of cochlear HCs, with some known cochlear HC markers being highly expressed, including *Gata3*, *Insm1*, *Hes6*, *Tmprss3*, and *Gng8*. Recently, Van der Valk *et al.* generated a comprehensive single-cell atlas of human inner ear tissues and inner ear-like organoids. By comparing with the human inner ear transcriptome, they elucidated the major cell types present in human inner ear-like organoids, including vestibular HCs, vestibular SCs, and epithelial cells. However, there is a relative scarcity of endolymphatic sac cells and cochlear cell types [43]. Shi *et al.* analyzed scRNA-seq of zebrafish inner ears from embryonic to adult stages and identified a putative progenitor cell population for all HCs and SCs in the inner ear using maker genes *Fgfr2*, *Fat1a*, *Igsf3*, and *Pard3bb* [44], as well as found that *Fgfr2* and *Igsf3* were downregulated in HCs and SCs but *Fat1a* and *Pard3bb* were downregulated in HCs only. Moreover, *Tectb* is expressed only in the maculae but *Zpld1a* in the cristae only.

Currently, scRNA-seq studies on auditory epithelial cell development are primarily focused on mice, with no reports on the joint analysis of single-cell atlases across multiple species. However, a study had localized human deafness genes in mice and found that the expression of human deafness genes in specific cell types of mice is consistent with reports in humans [31]. Another research based on scRNA-seq in zebrafish analyzed the expression of 42.86% of human non-syndromic hearing loss (NSHL) genes in zebrafish HCs, while more than 3000 genes still exhibited specific expression in zebrafish HCs [27]. We anticipate that future scRNA-seq studies considering inter-species comparisons will emerge, as this knowledge can provide valuable insights into the evolutionary conservation and divergence of auditory cell types.

### Auditory Epithelial Cell Damage and Disease

The genetic basis of age-related hearing impairment (ARHI) remains unclear at present [45]. Kalra *et al.* conducted a joint analysis of Genome-wide association study (GWAS) and multiple hearing-related feature data and single-cell transcriptome data from P2 mouse cochlea [46], finding that some fine-mapped risk genes were selectively expressed in HCs, including seven newly identified risk genes (*Ankra2*, *Ccdc68*, *Exoc6*, *Gnao1*, *Arhgef28*, *Crip3*, *Iqcb1* and *Klhdc7b*). Xue *et al.* used single-cell transcriptome data to find that genes associated with single-nucleotide polymorphisms (SNP) related to ARHI were widely expressed in varying cell types from the cochlea, while genes associated with the most significantly associated SNP were preferentially expressed in HCs (e.g., *Synj2*, *Cdh23*, *Ccdc68*) [47]. Remarkably, some functionally determined genes (e.g., *Mybpc3*, *Spi1*, *Slc39a13*) significantly overlapped with genes associated with Alzheimer's disease (AD), suggesting that the mechanism of ARHI may affect the risk of AD.

Sudden sensorineural hearing loss (SSNHL) is a prevalent auditory disease [48]. Nelson *et al.* screened 96 candidate SSNHL genes and mapped these genes to previously published datasets (GSE136196, GSE137299, GSE114157) [49], and found that SSNHL-related genes *Sod1*, *Hspa1a*, *Gpx1,* and *Mif* were enriched in IHCs and OHCs, pillar cells (PCs), and Deiters cells (DCs) at P7, and *Apoe*, *Gjb2,* and *Pon2* was also enriched in PCs and DCs at P7, but *Sod1*, *Gpx1*, *Mif,* and *Gjb2* were highly expressed in IHCs and DCs at P15.

Mutations of *Tmprss3* account for 9% of cases of genetically related hearing loss [50]. In mice, mutations in *Tmprss3* cause rapid damage to auditory HCs by P12 [51]. Tang *et al.* analyzed scRNA-seq data from D35 mouse inner ear organotypic cultures (equivalent to P12-P14 mouse inner ear) to find many DEGs involved in the interaction of Kcnma1 (*Acta1*, *Apoe*), 14-3-3- Epsilon (*Cdkn1c*, *Gsn*), Ca^2+^ binding proteins (*Cib2*, *Tnnc2*), Ca^2+^ regulatory factors (*Sln*) and the extracellular matrix (ECM) (*Ptgds*, *Otog*) after *Tmprss3* knockout, revealing its crucial role in intracellular Ca^2+^ homeostasis in HCs [52].

The Prestin protein encoded by *Slc26a5* is the molecular basis for OHC electromotility and is enriched on the membrane of OHCs [53]. Zheng *et al.* analyzed scRNA-seq data from the Prestin knockout (KO) mice OHC to find that numerous genes related to lipid metabolism and cell death pathways were significantly upregulated, especially s *Abca2*, *St6galnac,* and *Capn2* [54]. Liu *et al.* integrated single-cell short-read and long-read RNA sequencing technologies to study the cochlea [55]. They elucidated the functional role of a new transcript variant of Otoferlin (a crucial gene in IHCs), which is capable of translating into a functional protein isoform. Surprisingly, this isoform exhibited normal functionality compared to the canonical isoform but displayed reduced sustained exocytosis specifically in IHCs.

Immune cells in the cochlea respond to noise-induced damage [56]. Rai *et al.* used flow cytometry, immunostaining, and existing scRNAseq data [57] to demonstrate that both innate and adaptive immune system cell types exist in the cochlea, especially B, T, NK, and bone marrow cells (macrophages and neutrophils) are the major immune cells [58]. scRNA-seq analysis found that mRNA metabolism-related genes (e.g. *Sfpq*, *Hnrnpu,* and *Snrrnp48*) were significantly downregulated in OHCs in the mouse inner ear before and after noise-induced trauma [59].

Benkafadar and colleagues characterized the dynamic transcriptome during the apoptotic process of tall and short HCs of avian cochlear HCs using scRNA-seq data of the control group and the treatment group [60], finding that numerous genes were down-regulated in the first two types of HCs, while only 19 genes were up-regulated in tall HCs and 148 genes were instantly up-regulated in short HCs, especially commonly up-regulated genes are related to HC apoptosis (e.g., *Pim3*, *Abca8* and *Ywhag*). Remarkably, short HCs undergoing apoptosis displayed a specific expression pattern, with significantly up-regulated potassium channel genes (*Kcne1*, *Kcnq1*, *Kcnh7,* and *Kcnd2*) and significantly down-regulated genes (e.g., *Actb*, *Ak1,* and *Chrm2*), suggesting that dying short HCs may attempt to rescue the function of K^+^ channels.

### Auditory Epithelial Cell Regeneration and Therapy

Inducing auditory HC regeneration by specific TFs (e.g. *Atoh1*) is increasingly recognized as a feasible treatment strategy for hearing impairment [61, 62]. A study overexpressed *Atoh1* in DCs and PCs of the mouse cochlea and performed scRNA-seq at multiple developmental time points (P12, P26, P33) to investigate the possibility of directly converting SCs into HCs *in vivo* [57]. Their work identified 51 potential reprogramming TFs and found that *Atoh1* and *Pou4f3* may act as key reprogramming factors for SC to HC conversion, and the TF *Isl1* was confirmed as a synergistic reprogramming factor to promote *Atoh1*-induced HC regeneration. Especially, Sun *et al.* successfully transformed Prestin+ OHC-like cells *in vivo* by inducing the two key TFs (*Atoh1* and *Ikzf2*) in adult mouse cochlear SCs [40], further analyzed scRNA-seq data to uncover the upregulation of more than 700 OHC-specific genes (e.g. *Cib2*, *Calb1*, and *Lhfpl5*) and the downregulation of more than 300 SC-specific genes (e.g. *Tuba1b*, *Gjb2*, and *Rorb*) in the OHC-like cells, indicating that combined induction of *Atoh1* and *Ikzf2* is an effective pathway for SC regeneration and new OHC production. Iyer *et al.* used scRNA-seq to further compare the reprogramming potential of 3 different combinations of TFs (*Atoh1* alone, *Atoh1* + *Gfi1*, and *Atoh1* + *Gfi1* + *Pou4f3*) in the mouse cochlea [63]. Surprisingly, although the three combinations significantly improved the efficiency of HC reprogramming, the transcriptome of the resulting HCs was similar.

Quan *et al.* used scRNA-seq data to analyze the impacts of Myc/NICD activation on the reprogramming of adult mouse cochleae, finding that the interdental cells (IdC) have the largest impact among all the inner ear cell types [64]. Xia *et al.* also established a functional cochlear-like organ by initially reprogramming mouse cochlear precursor cells (CPCs) using various compounds and growth factors [65]. They use scRNA-seq data to further reveal the differentiation trajectory of CPCs and confirm the similarity to the early development trajectory of natural HCs, especially verifying that *Lgr5* expression was decreased gradually, while the expression of HC-specific genes e.g. *Myo6* and *Pou4f3*) increased. Interestingly, they also identified some specific TFs for periodic CPCs (*Cebpd*, *Lef1,* and *Myc*) and HCs (*Tal1*, *Zeb1*, and *Gata2*), and further validated two potential new HC markers *Acbd7* and *Ccer2*.

Previous study has demonstrated that activation of *Erbb2* in the cochlear SC subpopulation leads to a significant decrease in *Sox2* expression in adjacent cells, thereby increasing their proliferative and HC differentiation potential [66]. Piekna-Przybylska *et al.* compared *Erbb2*-activated SCs with inactive ones using scRNA-seq and discovered the formation of a new cell subpopulation within the *Erbb2*-activated SC subpopulation [67]. This subpopulation exhibited significant differentiation compared to the parental control cells, and widely expressed genes such as *Spp1* (a gene that responds to *Erbb2* signaling), *Mmp9*, *Timp1*, and *Dmp1*. Enrichment analysis indicated that these genes are involved in regulating ECM and cytokine responses.

The zebrafish otic vesicle has a similar inner ear structure to that of mammals, with the saccule having auditory functions [68], and the utricle primarily serving as the gravity sensor and exhibiting some auditory potential [69]. Each area has a thickened region called a macula, equivalent to the organ of Corti in mammalian inner ears, with the hair cells referred to as saccular macula hair cells (SMHCs) and utricular macula hair cells (UMHCs). The changes from the single-cell transcriptome during inner ear regeneration of zebrafish suggested that SCs may transition to an intermediate state of "progenitor cells" (significantly expressing *Dla*, *Her15.1,* and *Her4.1*), and then differentiate into HCs (significantly expressing *S100t*, *Pvalb8* and *Atoh1a*) in the zebrafish inner ear [70], and found that *Pax2a*, *Six1b*, *Notch3*, *Otogl1*, *Her6* (*Hes1* in mammals), and two HC marker genes (*Atoh1a* and *S100t*) were significantly increased in progenitor cells, as well as identified critical regulatory factors (*Atoh1a Her4.1* and *Cldn7b*) related to switch-like changes. Furthermore, through a multi-omics analysis of scATAC-seq and scRNA-seq, they further determined that the TFs *Sox* and *Six* regulatory networks are involved in the process of HC regeneration, especially validating the crucial role of the 2.6 kb regeneration-responsive enhancer upstream of the *Sox2* promoter in zebrafish auditory regeneration.

The auditory organ of birds, the basilar papilla, differs significantly from the mammalian cochlea in its ability to regenerate sensory HCs, allowing it to restore hearing within a few weeks following HC damage [71, 72]. To investigate the mechanism of HC regeneration in avian auditory organs, Janesick *et al.* analyzed the transcriptome changes in the chicken basilar papilla induced by aminoglycoside antibiotics (sisomicin) at single-cell resolution [73] and found that immune-related genes (*Ifi6*, *Ifit5*, and *Oasl*) associated with JAK/STAT pathway were significantly upregulated in SCs following HC damage, but were blocked by the inhibitor Ruxolitinib (RUX). The marker genes *Usp18* and *Calb2* were found to be shared by SCs and newly generated HCs. *Usp18* can inhibit JAK/STAT signaling, while *Calb2* is an unreported regeneration response, suggesting that JAK-STAT signaling may mediate the expression of immune-related genes in SCs of the chicken basilar papilla following HC damage, thus leading to the regeneration of new HCs. Matsunaga *et al.* also performed scRNA-seq on different stages of SC-to-HC conversion in the chicken basilar papilla explant culture [74], and identified a dynamic gene expression change in SCs, as well as divided the process into initiation, early, middle, and late stages, especially the upregulation of *Atoh1* and the transient upregulation of *Loxl1* and *Vim* were the features of the induction phase.

Gene therapy is currently the most promising approach for the treatment of genetic hearing loss [75]. RNA-Seq provides valuable insights into the design of gene therapy drugs and vectors for the treatment of hearing loss [34]. Moreover, by comparing single-cell transcriptomic profiles before and after gene therapy interventions, we can evaluate the efficacy of gene therapies at the single-cell resolution, thereby helping to optimize future gene therapy strategies. Iwasa *et al.* used woodchuck hepatitis virus post-transcriptional regulatory element (WPRE) to enhance *Tmc1* transgene expression to result in poor hearing recovery [76] and found that the expression of *Tmc1* in OHCs in animals treated with the WPRE-containing vector was higher than the group without WPRE through scRNA-seq analysis, with relatively better treatment effects, suggesting that optimizing the transgenic dose expressed in target cells is critical for gene therapy for hearing loss.

The studies on auditory sensory epithelium using scRNA-seq are summarized in Table 1.

**Table 1:** Application of scRNA-seq in auditory sensory epithelium.

Main idea	Year	Title	Journal	Species	Method	Data source
Auditory epithelial cell heterogeneity	2020	Characterizing Adult Cochlear Supporting Cell Transcriptional Diversity Using Single-Cell RNA-Seq Validation in the Adult Mouse and Translational Implications for the Adult Human Cochlea	Frontiers in Molecular Neuroscience	Mouse	Fluidigm C1	GSE135703
	2015	Single-cell RNA-Seq resolves cellular complexity in sensory organs from the neonatal inner ear	Nature Communications	Mouse	Fluidigm C1	GSE71982
	2019	Insights into the Biology of Hearing and Deafness Revealed by Single-Cell RNA Sequencing	Cell Reports	Mouse	SmartSeq2	GSE114157
	2015	Quantitative High-Resolution Cellular Map of the Organ of Corti	Cell Reports	Mouse	Fluidigm C1	N.A.
	2022	Single-cell RNA-sequencing of zebrafish hair cells reveals novel genes potentially involved in hearing loss	Cellular and Molecular Life Sciences	Zebrafish	10x Genomics	GSE221471
	2021	Cell-type identity of the avian cochlea	Cell Reports	Chicken	Smart-Seq2	SI
Auditory epithelial cell development and aging	2021	Mapping the regulatory landscape of auditory hair cells from single-cell multi-omics data	Genome Research	Mouse	10x Genomics	GSE157398, GSE157398
	2020	Characterization of the development of the mouse cochlear epithelium at the single-cell level	Nature Communications	Mouse	10x Genomics	GSE137299
	2022	Profiling mouse cochlear cell maturation using 10× Genomics single-cell transcriptomics	Frontiers in Cellular Neuroscience	Mouse	10x Genomics	GSE202920
	2022	Development and transdifferentiation into inner hair cells require Tbx2	National Science Review	Mouse	Smart-Seq2	GSE199369
	2018	Helios is a key transcriptional regulator of outer hair cell maturation	Nature	Mouse	10x Genomics	GSE120462
	2021	Cochlear Fibrocyte and Osteoblast Lineages Expressing Type 2 Deiodinase Identified with a Dio2CreERt2 Allele	Endocrinology	Mouse	Smart-Seq2	GSE181057
	2022	Critical roles of FGF, RA, and WNT signaling in the development of the human otic placode and subsequent lineages in a dish	Regenerative Therapy	Human inner ear organoid	10x Genomics	N.A.
	2023	Single-cell transcriptomic profiling of the zebrafish inner ear reveals molecularly distinct hair cells and supporting cell subtypes	eLife	Zebrafish	10x Genomics	GSE211728
	2023	Generating high-fidelity cochlear organoids from human pluripotent stem cells	Cell Stem Cell	Human inner ear organoid	10x Genomics	GSE200629
	2023	Single-cell transcriptomic profiling of the mouse cochlea: An atlas for targeted therapies	PNAS	Mouse	10x Genomics	umgear
	2023	A single-cell level comparison of human inner ear organoids with the human cochlea and vestibular organs	Cell Reports	Human and human inner ear organoid	10x Genomics	umgear
Auditory epithelial cell damage and disease	2020	Biological insights from multi-omic analysis of 31 genomic risk loci for adult hearing difficulty	PLOS Genetics	Mouse	10x Genomics	GSE135737
	2020	The immune response after noise damage in the cochlea is characterized by a heterogeneous mix of adaptive and innate immune cells	Scientific Reports	Mouse	–	–
	2021	Genes related to SNPs identified by Genome-wide association studies of age-related hearing loss show restriction to specific cell types in the adult mouse cochlea	Hearing Research	Mouse	10x Genomics	GSE181454
	2021	A cell-type-specific atlas of the inner ear transcriptional response to acoustic trauma	Cell Reports	Mouse	10x Genomics	GSE168041, GSE167078
	2022	In silico Single-Cell Analysis of Steroid-Responsive Gene Targets in the Mammalian Cochlea	Frontiers in Neurology	Mouse	–	–
	2021	Transcriptomic characterization of dying hair cells in the avian cochlea	Cell Reports	Chicken	Smart-Seq2	SI
	2019	Defective Tmprss3-Associated Hair Cell Degeneration in Inner Ear Organoids	Stem Cell Reports	Mouse inner ear organoid	10x Genomics	GSE130649
	2022	Prestin and electromotility may serve multiple roles in cochlear outer hair cells	Hearing Research	Mouse	Smart-Seq2	N.A.
	2023	Cochlear transcript diversity and its role in auditory functions implied by an otoferlin short isoform	Nature Communications	Mouse	10x Genomics and ScISOr-Seq	SRA: PRJNA759047
Auditory epithelial cell regeneration and therapy	2021	Dual expression of Atoh1 and Ikzf2 promotes the transformation of adult cochlear supporting cells into outer hair cells	eLife	Mouse	Smart-Seq2	GSE161156
	2022	Cellular reprogramming with ATOH1, GFI1, and POU4F3 implicates epigenetic changes and cell-cell signaling as obstacles to hair cell regeneration in mature mammals.	eLife	Mouse	10x Genomics	GSE182202
	2018	High-resolution transcriptional dissection of in vivo Atoh1-mediated hair cell conversion in mature cochleae identifies Isl1 as a co-reprogramming factor	PLOS Genetics	Mouse	10x Genomics	GSE85983
	2022	Avian auditory hair cell regeneration is accompanied by JAK-STAT-dependent expression of immune-related genes in supporting cells	Development	Chicken	Smart-Seq2	zenodo.5504624
	2022	Mutation-agnostic RNA interference with engineered replacement rescues Tmc1-related hearing loss	Life Science Alliance	Mouse	Smart-Seq2	umgear
	2022	A regulatory network of Sox and Six transcription factors initiate a cell fate transformation during hearing regeneration in adult zebrafish	Cell Genomics	Zebrafish	10x Genomics	GSE192947
	2021	TUB and ZNF532 Promote the Atoh1-Mediated Hair Cell Regeneration in Mouse Cochleae	Frontiers in Cellular Neuroscience	Mouse	–	–
	2022	Generation of innervated cochlear organoid recapitulates early development of auditory unit	Stem Cell Reports	Mouse	10x Genomics	GSE137299; GSE114157
	2023	Single-cell RNA sequencing analysis of mouse cochlear supporting cell transcriptomes with activated ERBB2 receptor indicates a cell-specific response that promotes CD44 activation	Frontiers in Cellular Neuroscience	Mouse	Smart-Seq2	GSE202850
	2023	Stepwise fate conversion of supporting cells to sensory hair cells in the chick auditory epithelium	iScience	Chicken	Fluidigm C1	GSE209791
	2023	Reprogramming by drug-like molecules leads to regeneration of cochlear hair cell-like cells in adult mice	PNAS	Mouse	10x Genomics	GSE205187

N.A., not available; SI, supporting information; –, no raw data was produced.

## Application of scRNA-seq in Cochlear SGN

### Cochlear SGN Heterogeneity

The sensitivity of Type I SGNs to sound and spontaneous firing rate (SR) varies [77]. Grandi *et al.* found three major subtypes of SGNI in the newborn mouse ear through scRNA-seq, which exclusively expressed *Lmx1a*, *Slc4a4,* and *Mfap4*/*Fzd2*, respectively [78]. Three studies used scRNA-seq to further investigate SGNs, each identifying three new subtypes of Type I neurons consistently, including Ia, Ib, and Ic neurons [79–81]. *Calb2*, *Pcdh20,* and *Rxrg* are highly expressed in Ia neurons, whereas *Calb1*, *Ttn,* and *Lrrc52* are enriched in Ib neurons, as well as *Runx1*, *Grm8* and *Lypd1* are enriched in Ic neurons. The diversity of SGN subtypes' transcription suggests significant physiological differences. Sun *et al.* found that genes related to mitochondrial function and neurofilaments (*Nefh*, *Nefm*, *Nefl*) were highly expressed in IA SGNs, corresponding to high-SR fibers [80]. One study showed that the voltage-gated Ca^2+^ channels genes *Cacna1b*, *Cacna1h* and *Cacna2d1* are enriched in Ia SGNs with the cholinergic receptor subunits *Chrna1* and *Chrna4*, and genes encoding Na^+^ channel *Nalcn*, K^+^ channel subunit *Cnip2* and *Kcnj9* are enriched in Ib and Ic SGNs, as well as *Drd1* (encoding dopamine receptor subunits) and *Grm8* (encoding metabotropic receptor subunits) are enriched in Ic SGNs [79].

Interestingly, Shrestha *et al.* also found that a portion of genes changed along the tonotopic axis in subtype-specific ways, including *Kcnip*, *Cpne6*, *Cacng5,* and *Pcdh9* [79]. Petitpre *et al.* found that neuronal diversity in the cochlea was established at birth by comparing single-cell RNA data from P3 newborn and mature mice [81]. Additionally, the scRNA-seq was used to compare nerve glial cells associated with the somatosensory dorsal root ganglia and auditory spiral ganglion [82] and found that *Gata2*, *Npy,* and *Epa3* specifically were expressed in the ear's GPs, especially *Npy* acts as a marker for the ear's GP.

### Cochlear SGN Development and Aging

Sun *et al.* analyzed the single-cell transcriptome from the early embryonic auditory nerve to find that auditory neurons had not yet differentiated into subtypes at E13.5, but highly expressed genes *Shox2*, *Myt1*, *Casz1,* and *Sall3* may be involved in the development of the inner ear ganglion [83]. Xu *et al.* further used scRNA-seq to identify the dynamic expression pattern of a new marker gene called *Miat* for SGNs [33]. Of note, Petitpré *et al.* used scRNA-seq to identify some temporally expressed genes (e.g., *Tle2*, *Pou4f1*, *Rbfox3*, *Id149*, *Gata3,* and *Neurod1*) involved potentially in fate determination of diverse SGNs through binary decisions [84]. Sanders and Kelley used scRNA-seq to further analyze SGNs throughout prenatal development and identified all four SGN subtypes (1A, B, C, and 2) that appeared at E18, as well as found that Immature SGNs initially divided into two precursor types (1A/2 type and 1B/C type) at E14 [85].

### Cochlear SGN Damage and Disease

Current studies suggest that corticosteroids may promote hearing impairment [86, 87]. Nelson *et al.* utilized previously published datasets (GSE152551, GSE137299, GSE114997) to locate steroid-responsive genes in various cell types of the cochlea, and found that *Nr3c2* and *Cacna1d* are broadly expressed in type 1 SGN cells [88], especially these steroid-responsive DEGs in SGN were related to cytokine-mediated anti-inflammatory pathways, suggesting that SGN is likely the main cellular type through which corticosteroids relieving inner ear inflammation, and some steroid-responsive DEGs (e.g., *Kcnh2*, *Grin1*, *Kcnt1* and *Cacna1a*) may be potential druggable gene targets.

Another study found that the SGN of mice exposed to noise underwent significant changes in transcriptional levels using scRNA-seq, indicating their involvement in the inner ear's adaptation process to sound stimulation, especially the ATF 3/4 pathway was strongly induced in 1A-type SGNs after noise exposure [59]. Of note, some key regulators and effectors (e.g., *Gadd45a*, *Ddit3,* and *Vgf*) were activated in response to noise. Genes specifically inhibited in 1A-type were enriched in synaptic genes, such as *Ank 2*, *Kcnc3*, *Rasgrp2*, *Grid2,* and *Grid1*.

The studies on cochlear SGN using scRNA-seq are summarized in Table 2.

**Table 2:** Application of scRNA-seq in cochlear SGN.

Main idea	Year	Title	Journal	Species	Method	Data source
Cochlear SGN heterogeneity	2020	Single-Cell RNA Analysis of Type I Spiral Ganglion Neurons Reveals a Lmx1a Population in the Cochlea	Frontiers in Molecular Neuroscience	Mouse	–	–
	2018	Hair Cell Mechanotransduction Regulates Spontaneous Activity and Spiral Ganglion Subtype Specification in the Auditory System	Cell	Mouse	10x Genomics	GSE114759
	2021	Diversity of developing peripheral glia revealed by single-cell RNA sequencing	Developmental Cell	Mouse	inDrops	GSE172110
Cochlear SGN development and aging	2022	Profiling mouse cochlear cell maturation using 10× Genomics single-cell transcriptomics	Frontiers in Cellular Neuroscience	Mouse	10x Genomics	GSE202920
	2022	Single-cell transcriptomic landscapes of the otic neuronal lineage at multiple early embryonic ages	Cell Reports	Mouse	10x Genomics	GSE178931
	2022	Single-cell RNA-sequencing analysis of the developing mouse inner ear identifies molecular logic of auditory neuron diversification	Nature Communications	Mouse	Smart-seq2	GSE165502
	2022	Specification of neuronal subtypes in the spiral ganglion begins prior to birth in the mouse	PNAS	Mouse	10x Genomics	GSE195500
Cochlear SGN damage and disease	2021	A cell-type-specific atlas of the inner ear transcriptional response to acoustic trauma	Cell Reports	Mouse	10x Genomics	GSE168041
	2022	In silico Single-Cell Analysis of Steroid-Responsive Gene Targets in the Mammalian Cochlea	Frontiers in Neurology	Mouse	–	–
	2022	Repurposable Drugs That Interact with Steroid Responsive Gene Targets for Inner Ear Disease	Biomolecules	Mouse	–	–

N.A., not available; SI, supporting information; –, no raw data was produced.

## Application of scRNA-seq in Other Types of Inner Ear Cells

### Otic Vesicle (OV)

During the early embryonic development, the inner ear organ undergoes an invagination from the otic placode, followed by a separation from non-neural ectoderm (NNE) through the pre-placodal ectoderm (PPE) to form the otic vesicle (OV) [89]. A study performed scRNA-seq on the OV of normal and *Tbx2*/*3c*KO embryos and identified a multipotent population of otic progenitor cells in normal OVs that was marked by *Eya1*, *Sox2*, *Sox3*, *Fgf18*, *Cxcl12* and *Pou3f3* [90]. In *Tbx2*/*3*cKO embryos, the number of otic progenitor cells increased by three-fold, but their genes were dysregulated (e.g. *Dlx5*, *Otx2*, *Wnt2b*, *Gli1*). Interestingly, ectopic immature neuronal clusters (*Neurog1* and *Neurod1*) were detected in the OVs of *Tbx2*/*3*cKO, implying that *Tbx2/3* loss may lead to delayed maturation, further disrupting the morphogenesis of the OV.

Moreover, the OV is the origin of the vast majority of inner ear cells, such as auditory epithelial cells and neurons. Durruthy-Durruthy *et al.* analyzed scRNA-seq data to identify different cell populations and found that the downregulation of Shh and Wnt signaling ensures the development of neuronal lineage, and specific Notch genes (*Foxgl* and *Jag2*) may be involved in the later development of auditory and vestibular ganglia [91]. Interestingly, Notch signaling is mainly localized in dorsal and anterior cells (*Notch2*) and ventral anterior cells (*Hey1*, *Hes1*, and *Hey2*), while Shh signaling is primarily concentrated in ventral OV cells (*Ptc2* and *Gli3*). Especially, the precursor cells identified in the ventral-associated cells express some genes in the Notch (*Hey1*, *Hey2*, *Hes1*), Shh (*Gli2* and *Gli3*) and Fgf (*Fgf3* and *Fgf10*) pathways, indicating that the interplay among Notch, Shh and Fgf signaling may induce the establishment of the sensory lineage. Buzzi *et al.* further performed a joint analysis of scRNA-seq and ATAC-seq data from otic-epibranchial progenitors (OEPs) after NNE and identified the key components of the ear specification network: *Sox8*, *Pax2,* and *Lmx1a*, especially *Sox8* may control ear fate [92].

Eli *et al.* manipulated FGF, Wnt, BMP, and RA signaling pathways in human embryonic stem cells, and induced pluripotent stem cells to guide the differentiation of NNE, as well as utilized scRNA-seq to detect the expression of NNE markers *Dlx6*, *Dlx5,* and *Gata3* followed by the appearance of PPE and OV markers *Eya1*, *Six4*, *Eya2*, *Myosin7a*, *Foxi3* and *Fbxo2* [93]. They also found that the transcriptional profile of cells on D12 of the induction process was most similar to that of E10.5 mouse OV cells, but further development seemed to be hindered. Another study explored the impact of Wnt signaling on early PPE differentiation in stem cell-derived organoids of the inner ear using scRNA-seq and found that Wnt signaling significantly influenced the efficiency of inner ear organoid induction and showed similarity to *in vivo* models [94].

Mutations of the *Chd7* gene lead to malformations in the inner ear and severe sensorineural hearing impairment [95–97]. Analyzing single-cell transcriptome from E10.5 mouse OVs lacking *Chd7* and qRT-PCR experiment found that the expression of pro-neural (e.g., *Neurod1*, *Neurog1*) and Notch-related genes (e.g., *Hes5*, *Hey1*) was dysregulated in the ventral OV [98]. Nie *et al.* utilized *Chd7*-mutant human induced pluripotent stem cell-derived organoids as a model and identified failed generation of HCs and SCs through scRNA-seq [99]. They found that numerous deafness-related genes (e.g. *Col9a2*, *Lmx1a,* and *Sox10*) were downregulated, and single-cell transcriptome data from *Chd7*KO/+ organoids also showed that some deafness-related genes (e.g. *Six1*, *Ush1c,* and *Strc*) were downregulated in HCs, implying that. The dysregulated expression of deafness-related genes due to *Chd7* mutations may result in hearing loss observed in CHARGE syndrome patients.

### Greater Epithelial Ridge (GER)

GER cells are a short-lived group of newly formed cells in the cochlear duct. Analyzing scRNA-seq data from the cochlear duct of newborn rats on P1 and P7 found that four cell groups had significantly reduced cell numbers, and many DEGs were enriched in the ribosome and PI3K-Akt pathways, especially *Rps16*, *Rpsa*, *Col4a2*, *Col6a2*, *Ctsk* and *Jun* were highly expressed, which may be involved in GER degeneration during normal development [100].

A previous study found that the GER region can produce a specific marker *Myosin7a* for HCs [101]. Subsequently, Kubota *et al.* used scRNA-seq to find HC-positive cells (*Myosin7a*) in GER cell-derived organoids and identify three major cell subtypes of P2 GER cells: inner (*Calb1*, *Epyc*, *Crabp1*), middle (*Ccdc3*, *Msc*, *Penk*) and outer (*Rorb*, *Thbs1*, *Lgr5*) [102]. They found that the neonatal cochlear stem cell gene (*Lgr5*) was highly expressed in outer GER cells, suggesting that GER cells may be a precursor cell pool for cochlear HC regeneration. Chen *et al.* further identified eight subtypes of GER cells through scRNA-seq, four of which continuously increased in cell number from P1 to P7 but incrementally decreased and disappeared at P14, and all highly expressed genes were related to hearing loss, such as *Col2a1*, *Col9a1*, *Col9a2* and *Col11a2*, especially two of these subtypes mainly differentiated into OHCs with highly expressed genes *Crym*, *Col9a2*, *Col2a1*, *Col9a1*, *Cytl1*, *Cnmd*, *Serpinf1,* and *Col11a2*, respectively, but other two subtypes (*Lepr*, *Ramp3*; *Igfbp4*) ultimately differentiated into IHCs [103].

Kempfle *et al.*'s work suggests that HCs develop from embryonic Sox2+ progenitor cells [104]. Using this published single-cell data [31], Xu *et al.* found that the Sox2+ cell population in the P1 stage mainly comprised DCs, IPhCs, and lateral GER cells [105]. Their work suggested that the Sox2+ outer GER may be the most amenable to electroporation and cause the least damage to HC. The single-cell transcriptomic data from *Atoh1*+*Gfi1*+*Pou4f3*-induced mouse cochlear cells also showed that GER cells harbor a large number of reprogrammed cells with HC-like gene features and GLAST+ and SOX2+ reprogramming-SCs, and Notch receptor genes (*Lfng*, *Notch1*, and *Hes1*) were upregulated in the reprogramming-SCs, while Notch ligand genes specific to HCs (*Dll3*, *Jag2*, and *Dlk2*) were upregulated in the reprogrammed HCs [63]. These findings imply that TF reprogramming may possess the ability to redefine the interactions of the Notch signaling between HCs and SCs, thereby inhibiting the reprogramming of SCs surrounding the reprogrammed HCs.

### Stria Vascularis (SV)

The generation and maintenance of the vital endocochlear potential (EP), which is necessary for the acoustic transduction and auditory activity of cochlear HCs, is facilitated by the stria vascularis (SV), located on the lateral aspect of the cochlear wall. However, SV is a complex heterogeneous tissue, especially its’ cellular composition is not fully understood. Korrapati *et al.* used scRNA-seq and single-nucleus RNA-seq to analyze the cell composition of mature mouse (P30) SV [106] and identified new cell type-specific genes, such as *Abcg1* and *Heyl* in marginal cells, *Nrp2* and *Kcnj13* in intermediate cells, *Sox8* and *Nr2f2* in basal cells, and *P2rx2* and *Kcnj16* in spindle/root cells. Specifically, they validated the co-expression of *Esrrb* enhancer and its direct target genes *Abcg1*, *Heyl,* and *Atp13a5* in SV marginal cells. Interestingly, the *Bmyc* enhancer and its direct target genes *Cd44*, *Met,* and *Pax3* were also validated to be co-expressed in SV intermediate cells. Moreover, Gu *et al.* further identified *Lgr5* and *Epyc* as marker genes for root cells, while *Anxa1* and *Dpp10* were specifically expressed in spindle cells and confirmed by smFISH [107].

Studies found that cisplatin can cause long-term accumulation within SV in both humans and mice, thereby leading to a decrease in EP in the cochlea [108, 109]. Two studies compared the scRNA-seq dataset from the adult SV with cisplatin-induced ototoxicity and the normal adult SV in mice [106, 110]. They found that basal cells had few DEGs, while the edge and intermediate cells of the SV were significantly affected by cisplatin treatment. EP-related genes (*Kcnj10*, *Gjb2*, *Met*) were downregulated in intermediate cells of the cisplatin-induced ototoxicity mouse model, and EP-related genes (*Kcne1*, *Atp1b2,* and *Kcnq1*) specific to the marginal cells of the SV were downregulated, as well as marginal cell-specific regulator *Klf10* and intermediate cell-specific regulator *Tbx2* were identified. Additionally, the Ionic homeostatic function of the SV may be affected by MD [111, 112]. Gu *et al.* located genes associated with MD to different cell types of the adult mouse SV using the original single-cell transcriptome dataset and found that MD-associated genes *Atp1b2* and *Kcne1* were located in the marginal cells of the SV, while MD-associated genes *Met* and *Ednrb* in the intermediate cell layer of the SV [106, 113].

One study compared single-cell transcriptome data from the majority of cell types in the SV of mice between a noise-induced group and a normal control group [59]. They found that most cell types in the SV showed significant downregulation of potassium ion transport-related genes under noise-induced injury. To further understand the mechanism of cochlear aging related to ARHL, Sun *et al.* characterized the dynamic single-cell transcriptome landscape of mouse cochlear aging at five different time points [114]. They found significant transcriptional changes in intermediate cells of the SV during aging, where protein homeostasis loss and cell apoptosis were most prominent, especially the elevated level of the chaperone protein *Hsp90aa1* during aging could mitigate damage caused by endoplasmic reticulum stress in SV cells, implying that this mechanism of compensation may alleviate SV atrophy caused by aging, thereby slowing the progression of ARHL. Nelson *et al.* screened 96 candidate genes for SSNHL and found a high expression of SSNHL-related genes, such as *Sod1*, *Gpx3,* and *Mif* in SV cell types [49].

Of note, using the published single-cell transcriptomics data after hearing damage [59] in conjunction with whole-genome meta-analysis, Trpchevska *et al.* identified some important contributors to hearing loss in the SV spindle cells and basal cells [115]. Among them, *Eya4*, *Homer2,* and *Gas2* were identified in the SV spindle cells that were associated with SNPs of GWAS significance, while *Nid2*, *Pc*, *Ccs,* and *Ahdc1* were identified in the basal cells.

### Endolymphatic Sac

The inner ear's endolymphatic sac is an evolutionarily conserved structure that is essential for shaping the morphology and developmental progression of hearing function [116]. A recent study analyzed the scRNA-seq data from the endolymphatic sac before and after birth [117], and found that early-stage ribosome-rich cells (RRCs) in the endolymphatic sac express genes (*Lmx1a*, *Dach2,* and *Bmp3*) associated with extracellular protein expression and secretion, while mature RRCs exhibit the expression of genes (*Lcn2*, *Slpi,* and *Serping1*) associated with innate immunity, especially mitochondria-rich cells (MRCs) have high expression of genes (*Slc26a4*, *Clcnkb,* and *Slc4a9*) that mediate ion transport, suggesting that MRCs may have an important function in the reabsorption of endolymphatic fluid dependent on *Slc26a4*.

Heterozygous mutations in *Sox9* can cause both hearing loss and campomelic dysplasia (CD) [118]. A mouse model confirmed that mutations in *Sox9* affect the endolymphatic sac, thereby resulting in hearing loss [119]. Szeto *et al.* further investigated the underlying mechanisms by performing scRNA-seq on endolymphatic sacs from WT and *Sox9* mutant mice [120]. Firstly, they found an increased proportion of immature cells and a decreased proportion of mature cells in the mutants. Secondly, they observed abnormal regulation of genes involved in fluid regulation (*Slc24a4*, *Slc15a1*, and *Ttyh1*) and Wnt signaling (*Dkk3*, *Ccnd2*, and *Wnt6*) in the RRCs of the mutants. Thirdly, SCENIC revealed that *Sox9* mutations further affect the activity of *Sox10* and its transcription targets (*Oc90*, *Dkk3*, and *Nox3*) in RRCs, which are critical for endolymphatic sac/inner ear function.

The studies on other cell types of inner ear cells using scRNA-seq are summarized in Table 3.

**Table 3:** Application of scRNA-seq in other types of inner ear cells.

Main idea	Year	Title	Journal	Species	Method	Data source
Otic vesicle	2014	Reconstruction of the Mouse Otocyst and Early Neuroblast Lineage at Single-Cell Resolution	Cell	Mouse	Fluidigm C1	N.A.
	2016	Single-cell analysis delineates a trajectory toward the human early otic lineage	PNAS	Human	Fluidigm C1	N.A.
	2018	Single Cell Transcriptomics Reveal Abnormalities in Neurosensory Patterning of the Chd7 Mutant Mouse Ear	Frontiers in Genetics	Mouse	Fluidigm C1	N.A.
	2023	Tbx2 and Tbx3 regulate cell fate progression of the otic vesicle for inner ear development	Developmental Biology	Mouse	10x Genomics	GSE185172
	2022	Early Wnt signaling activation promotes inner ear differentiation via cell caudalization in mouse stem cell-derived organoids	Stem Cells	Mouse	10x Genomics	GSE180062
	2022	Sox8 remodels the cranial ectoderm to generate the ear	PNAS	Chicken	Smart-seq2	GSE168089
Greater epithelial ridge	2021	Single-Cell RNA Sequencing Analysis Reveals Greater Epithelial Ridge Cells Degeneration During Postnatal Development of Cochlea in Rats	Frontiers in Cell and Developmental Biology	Rat	10x Genomics	GSE170810
	2022	Pseudo-Temporal Analysis of Single-Cell RNA Sequencing Reveals Trans-Differentiation Potential of Greater Epithelial Ridge Cells Into Hair Cells During Postnatal Development of Cochlea in Rats	Frontiers in Molecular Neuroscience	Rat	10x Genomics	GSE195702
	2021	Greater epithelial ridge cells are the principal organoid-forming progenitors of the mouse cochlea	Cell Reports	Mouse	Smart-seq2	GSE162308
	2022	Cellular reprogramming with ATOH1, GFI1, and POU4F3 implicates epigenetic changes and cell-cell signaling as obstacles to hair cell regeneration in mature mammals.	eLife	Mouse	10x Genomics	GSE182202
	2021	TUB and ZNF532 Promote the Atoh1-Mediated Hair Cell Regeneration in Mouse Cochleae	Frontiers in Cellular Neuroscience	Mouse	–	–
Stria vascularis	2022	Single-cell transcriptomic Atlas of mouse cochlear aging	*Protein & Cell*	Mouse	10x Genomics	GSA: CRA004814
	2021	A cell-type-specific atlas of the inner ear transcriptional response to acoustic trauma	Cell Reports	Mouse	10x Genomics	GSE168041; GSE167078
	2020	Characterization of rare spindle and root cell transcriptional profiles in the stria vascularis of the adult mouse cochlea	Scientific Reports	Mouse	10x Genomics	GSE152551
	2019	Single Cell and Single Nucleus RNA-Seq Reveal Cellular Heterogeneity and Homeostatic Regulatory Networks in Adult Mouse Stria Vascularis	Frontiers in Molecular Neuroscience	Mouse	10x Genomics	GSE136196
	2021	Single-Cell RNA-Seq of Cisplatin-Treated Adult Stria Vascularis Identifies Cell Type-Specific Regulatory Networks and Novel Therapeutic Gene Targets	Frontiers in Molecular Neuroscience	Mouse	10x Genomics	GSE165662
	2021	Identification of Potential Meniere's Disease Targets in the Adult Stria Vascularis	Frontiers in Neurology	Mouse	–	–
	2021	Utilizing Single Cell RNA-Sequencing to Implicate Cell Types and Therapeutic Targets for SSNHL in the Adult Cochlea	Otology & Neurotology	Mouse	–	–
	2022	Genome-wide association meta-analysis identifies 48 risk variants and highlights the role of the stria vascularis in hearing loss	The American Journal of Human Genetics	Mouse	–	–
Endolymphatic sac	2017	Molecular architecture underlying fluid absorption by the developing inner ear	elife	Mouse	Fluidigm C1	GSE87293
	2022	SOX9 and SOX10 control fluid homeostasis in the inner ear for hearing through independent and cooperative mechanisms	PNAS	Mouse	Smart-seq2	GSE131196, GSE139587

N.A., not available; SI, supporting information; –, no raw data was produced.

## Future Prospects and Conclusion

Although many features of scRNA-seq technology have brought unprecedented progress in the field of auditory research, there is still room for optimization and development. Of note, a novel scRNA-seq technology FIPRESCI has been developed, which has optimized the mainstream microfluidic platform and increased cell throughput in more than tenfold ways, especially greatly reduced costs [121], Meanwhile, a high-throughput, low-cost, and efficient single-cell temporal transcriptome sequencing method Well-TEMP-seq has also been developed, which can simultaneously resolve the expression dynamics of thousand genes of single cells [122].

The majority of commonly employed scRNA-seq techniques which utilize short-read sequencing methods exhibit limited precision in quantifying RNA at the allele and isoform resolution levels. Conversely, long-read sequencing technologies lack the depth necessary for large-scale cross-cell applications [123]. Consequently, full-length scRNA-seq has gained the capability to capture complete transcript sequences using two predominant strategies employed in this field: Smart-seq 3 [124] and long-read scRNA-seq [125]. Smart-seq 3 represents an advancement over its precursor, Smart-seq 2. It directly assigns individual RNA molecules to their respective isoforms and establishes their allelic origins within single cells. Subsequently, an enhanced version of Smart-seq 3, named Smart-seq3xpress, streamlines the time-consuming and costly library construction steps, diminishes reaction volumes, reduces expenses, and boosts sample throughput [126]. Another technique, namely single-cell isoform RNA-Seq (ScISOr-Seq), merges high-throughput single-cell capture platforms with conventional Iso-Seq, enabling the simultaneous full-length transcriptome sequencing of tens of thousands of cells. However, cost constraints have hindered large-scale single-cell sequencing (only 12 OHCs were sequenced) [26]. Recently, Liu *et al.* pioneered the integration of ScISOr-Seq with short-read scRNA-seq technology in cochlear research [55]. They identified over 120,000 novel transcripts, of which 90.14% exhibited coding potential. By examining these full-length transcripts and transcript isoforms, ScISOr-Seq enhances our comprehension of selective splicing events and isoform diversity in the cochlea, thereby expanding our understanding of the intricate regulatory networks underlying auditory function at the isoform resolution level.

Clearly, scRNA-seq also trends to integrate with other types of single-cell sequencing or third-generation sequencing. For example, the DMF-DR-seq method can simultaneously detect single-cell genome and transcriptome at the single-cell level [127]. The scPCOR-seq can simultaneously analyze RNA expression levels and chromatin occupancy of chromatin-binding proteins or histone modifications in the same single cell [128]. The SCAN-seq2 is a high-throughput and high-sensitivity full-length scRNA-seq analysis technology, which has high accuracy in distinguishing different RNA subtypes of the same gene at single-cell resolution [129].

Recently, some technologies related to accurately capturing the spatiotemporal information of single cells have made breakthroughs, which will provide help for revealing auditory organ development and lesion progression. The Live-seq is a live-cell transcriptome sequencing technology, which can enable transcriptome analysis of the same cell at different time points while maintaining cell viability [130]. Additionally, some spatial transcriptomics technologies (e.g. MERFISH, Slide-seq, and Stereo-seq) further compensate for the spatial information [131]. The combination of spatial transcriptome and scRNA-seq can effectively understand the relationship between gene expression and morphology in single cells and the local environment.

**Fig. 3:**
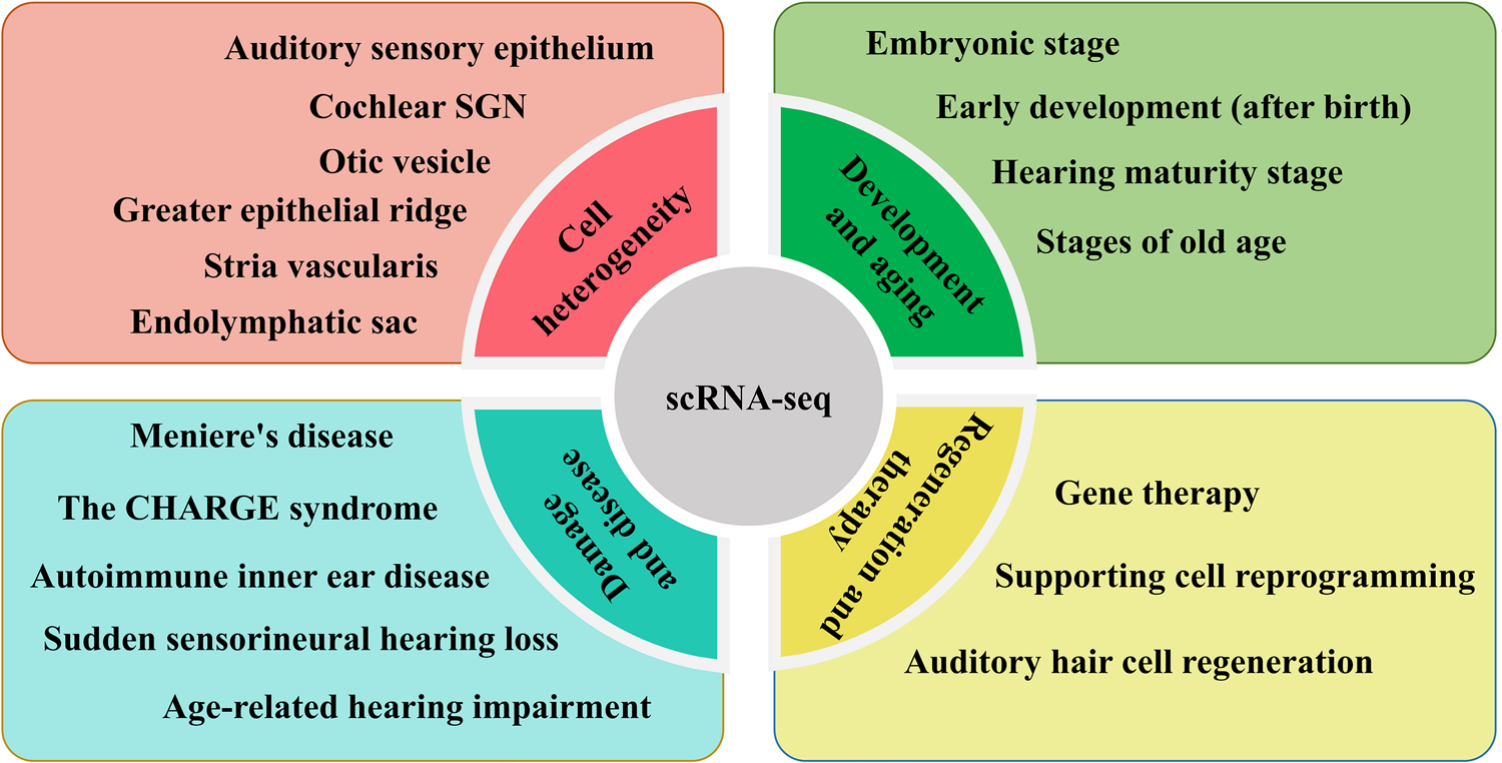
Hearing loss and deafness have been the subject of global concern as they are prevalent disabilities. Through the utilization of scRNA-seq, many aspects in the field of auditory research have been significantly advanced, including the heterogeneity, development, aging, and deafness-related diseases of auditory organ cells, as well as the regeneration and treatment of hair cells.

Overall, the scRNA-seq technology has been widely used in the field of inner ear hearing (Fig. 3) and has made significant contributions to the study of different roles that various cell types play in the development and hearing loss of auditory organs. It provides not only single-cell-accurate transcriptome information for the treatment of hair cell regeneration and hearing recovery but also more dimensional information for the auditory organ with the other technologies mentioned above as well as knowledge reserved for related clinical applications in the future.
